# Unveiling molecular mechanism of ET‐1 induced vascular insult through RNAseq analysis

**DOI:** 10.1002/alz.088910

**Published:** 2025-01-03

**Authors:** Mayank Gupta, Ankita Talukdar, Rehab Hussain, Latha Diwakar

**Affiliations:** ^1^ Centre for Brain Research, Indian Institute of Science, Bengaluru, Karnataka India; ^2^ Manipal Academy of Higher Education, Manipal, Karnataka India

## Abstract

**Background:**

Vascular Dementia (VaD) is the second most prevalent cause of dementia, arising from the blockage of blood vessels in the brain. One event responsible for the blockage or narrowing of small blood vessels is transient ischemic attack (TIA), and these changes resolve within 24 hours in humans. The molecular mechanism underlying these changes in recovery in small vessels still needs to be investigated. To address this gap, we have developed a mouse model by administering endothelin‐1 (ET‐1) into the lateral ventricles. This disrupts small vessel integrity, which leads to learning and memory deficits after 3 days of a single ET‐1 injection. Intriguingly, there is a subsequent recovery in these deficits after 30 days, mimicking the clinical scenario of TIAs. Bulk RNA sequencing from microvessels was performed to elucidate further the factors involved in recovery mechanisms.

**Method:**

ET‐1 injection was given to 4 groups of C57BL6/J mice, and mice were sacrificed at different time points of 3, 15, and 30 days along with saline counterparts. Microvessels were extracted from the pooled cortex and hippocampus, followed by RNA isolation. RNA samples were subjected to the cDNA library preparation, and RNA sequencing was conducted using the Illumina NovaSeq 6000® instrument. Transcriptomic analysis was carried out in the Linux environment. Differential gene expression was performed using R.

**Result:**

We observed biological processes such as vasculature development, angiogenesis, and wound healing start upregulating after 3 days of a single ET‐1 injection, whereas several processes involved in ion transport chains were downregulated. Further, biological processes for muscle development, mitochondrial electron transport chain, and neuronal development were upregulated after 15 days of a single ET‐1 injection. No biological processes were affected after 30 days of a single ET‐1 injection, suggesting a possible complete recovery from ET‐1‐mediated vascular insult by the 30th day.

**Conclusion:**

Our results highlight the temporal dynamics of biological processes in response to a single ET‐1 injection. It offers valuable insight for understanding the mechanism behind the recovery and factors involved in this process after vascular insult, paving the way for therapeutic targets.

